# The Impact of COVID-19 Vaccine Communication, Acceptance, and Practices (CO-VIN-CAP) on Vaccine Hesitancy in an Indian Setting: Protocol for a Cross-sectional Study

**DOI:** 10.2196/29733

**Published:** 2021-06-24

**Authors:** Krishna Mohan Surapaneni, Mahima Kaur, Ritika Kaur, Ashoo Grover, Ashish Joshi

**Affiliations:** 1 Panimalar Medical College Hospital & Research Institute Chennai India; 2 SMAART Population Health Informatics Intervention Center Foundation of Healthcare Technologies Society Panimalar Medical College Hospital & Research Institute Chennai India; 3 Foundation of Healthcare Technologies Society Delhi India; 4 Indian Council of Medical Research Delhi India; 5 City University of New York Graduate School of Public Health and Health Policy New York, NY United States

**Keywords:** COVID-19 vaccine, vaccine hesitancy, vaccine acceptance, unintended consequences, vaccination, COVID-19, pandemic, coronavirus, infectious disease, protocol, vaccine

## Abstract

**Background:**

COVID-19 vaccines are considered to be a key to limiting and eliminating the infectious disease. However, the success of the vaccination program will rely on the rates of vaccine acceptance among the population.

**Objective:**

This study aims to examine the factors that influence vaccine hesitancy and vaccine acceptance, and to explore the unintended consequences of COVID-19 infections. The study will further explore the association between sociodemographic characteristics; health status; COVID-19–related knowledge, attitude, and practices; and its influence on vaccine hesitancy and acceptance among individuals living in urban and rural settings of Chennai, Tamil Nadu in the southern state of India.

**Methods:**

A cross-sectional study will be conducted between January 2021 and January 2023. A sample of approximately 25,000 individuals will be recruited and enrolled using a nonprobability complete enumeration sampling method from 11 selected urban and rural settings of Chennai. The data will be collected at one time point by administering the questionnaire to the eligible study participants. The collected data will be used to assess the rates of vaccine acceptance; hesitancy; and knowledge, attitudes, practices, and beliefs regarding COVID-19 and COVID-19 vaccines. Lastly, the study questionnaire will be used to assess the unintended consequences of COVID-19 infection.

**Results:**

A pilot of 2500 individuals has been conducted to pretest the survey questionnaire. The data collection was initiated on March 1, 2021, and the initial results are planned for publication by June 2021. Descriptive analysis of the gathered data will be performed using SAS v9.1, and reporting of the results will be done at 95% CIs and *P*=.049. The study will help explore the burden of vaccine acceptance and hesitancy among individuals living in urban and rural settings of Chennai. Further, it will help to examine the variables that influence vaccine acceptance and hesitancy. Lastly, the findings will help to design and develop a user-centered informatics platform that can deliver multimedia-driven health education modules tailored to facilitate vaccine uptake in varied settings.

**Conclusions:**

The proposed study will help in understanding the rate and determinants of COVID-19 vaccine acceptance and hesitancy among the population of Chennai. The findings of the study would further facilitate the development of a multifaceted intervention to enhance vaccine acceptance among the population.

**International Registered Report Identifier (IRRID):**

DERR1-10.2196/29733

## Introduction

### Background and Rationale

The COVID-19 pandemic continues to impose burdens of morbidity and mortality while disrupting societies and economies worldwide since 2020. These negative impacts motivated pharmaceutical companies to develop a vaccine immediately. Vaccination of people against COVID-19 has now started in many countries [1]. Governments prepare themselves to ensure large-scale equitable access and distribution of safe and effective COVID-19 vaccines. This will require sufficient health system capacity and effective strategies to increase trust in vaccines and those who deliver them [2]. For decades, vaccines have been a successful measure to eliminate and prevent numerous infections. However, concern about vaccine hesitancy is growing worldwide, prompting the World Health Organization (WHO) to declare it among the top 10 health threats in 2019 [3]. In 2015, the WHO Strategic Advisory Group of Experts on Immunization defined vaccine hesitancy as a “delay in acceptance or refusal of vaccination despite the availability of vaccination services” [4]. In many countries, vaccine hesitancy and misinformation present substantial obstacles to achieving a high coverage rate necessary to attain herd immunity to flatten the epidemic curve [5,6]. Several determinants influence whether an individual refuses, delays, or accepts some vaccines. These include historical, socioeconomic, cultural, ecological, health system/institutional, and political factors [7].

Governments, public health officials, and advocacy groups must be prepared to address the issue of vaccine hesitancy and lower vaccine acceptance rates [2]. Misinformation regarding the benefits, medicinal composition, and adverse effects of vaccination spread through multiple channels could have a considerable effect on the acceptance and increased COVID-19 vaccine hesitancy [8]. Effective interventions should directly address community-specific concerns or misconceptions and be sensitive to religious or cultural beliefs [9]. Trust in government is strongly associated with vaccine acceptance and can contribute to public compliance with recommended actions [10]. Clear and consistent communication by government officials is central in building confidence in vaccine programs among individuals. This includes explaining the development of vaccines, how it works, and its safety and efficacy. Campaigns should also aim to explain a vaccine’s level of effectiveness, the time needed for attaining protection, and the importance of population-wide inoculation with the COVID-19 vaccine to achieve herd immunity. Health communication must reach all communities to enhance vaccine literacy to prevent future infections and mortality [11,12]. Effective and strategic health messages are one of the key approaches in assisting higher authorities to deal with increased vaccine hesitancy and to slow the spread of the infection. Improving vaccine uptake among those most hesitant will be of utmost importance in reaching the immunization rates needed for community immunity [13,14].

Further, some unintended consequences have emerged since the inception of the COVID-19 pandemic, such as lifestyle and behavior changes, impacts on mental health, and economic consequences. These consequences are likely to increase over time as the multiple waves of the COVID-19 pandemic continue to develop [15].

India, being one of the most populated countries in the world, has been struggling to attain the goal of 90% vaccine coverage under the national immunization schedule due to several reasons including vaccine hesitancy [16,17]. According to the National Family Health Survey (NFHS) 1 (1992-1993), there was 65% vaccine coverage, which increased to 82% in NFHS 2 (1998-1999). This was followed by 81% as per NFHS 3 (2005-2006) to a substantial decline to 69.7% as per the NFHS 4 (2015-16) in Tamil Nadu [18]. In the region of Chennai, Tamil Nadu, a southern state in India, a considerable decline in the vaccine coverage rate has been observed over the past 20 years [19,20].

The government of India has now opened up COVID-19 vaccinations for everyone 18 years and older. Until May 8, 2021, 169,439,663 total doses were administered, out of which 133,980,544 received the first dose and 35,459,119 were administered the second dose. Meanwhile, in Tamil Nadu to date, 6,480,287 have been vaccinated, of which 4,835,514 received the first dose and 1,644,773 received the second dose. As of May 8, 2021, Tamil Nadu stands 10th in India in administering total vaccine doses. Tamil Nadu recorded a decline in the vaccination rate between April 1 to 10, 2021, compared to March 22 to 29, 2021. The vaccination coverage once again recorded a dip on April 18, 2021. On April 18, 41,120 were newly vaccinated as compared to 138,298 the previous day. Even though the new cases in Tamil Nadu are rising and the vaccinations are open for individuals 18 years and older, the new vaccinated rate is low and inconsistent [21]. This reduction and inconsistency in the vaccine coverage rates could be due to vaccine hesitancy among individuals. There also seems to be a paucity of data on the rate of COVID-19 vaccine acceptance, vaccine hesitancy, and factors contributing to them. To control vaccine preventable diseases, it is imperative to identify the key challenges and opportunities to reduce vaccine hesitancy and recoup vaccine confidence among individuals. Hence, this study aims to examine the factors that influence vaccine hesitancy and vaccine acceptance, and to explore the unintended consequences of COVID-19 infections. The study will further explore the association between sociodemographic characteristics; health status; COVID-19–related knowledge, attitude, and practices; and its influence on vaccine hesitancy and acceptance among individuals living in urban and rural settings of Chennai District of Tamil Nadu, a southern state in India.

### Study Objectives

This study aims to assess the rates of vaccine acceptance and vaccine hesitancy among the adult population of Chennai, Tamil Nadu; to investigate the determinants of vaccine acceptance and vaccine hesitancy; to explore the unintended consequences of COVID-19 infection; and to formulate an evidence-driven intervention model to facilitate uptake of the COVID-19 vaccine in Chennai and other states of India.

## Methods

### Study Design and Population

A cross-sectional study will be conducted between the period of January 2021 and January 2023. The study participants will be recruited from 11 selected urban and rural settings of Chennai District of Tamil Nadu, a southern state in India. A total of 11 primary health centers (PHCs) of Chennai District were selected for the study, namely, Thiruninrarvur, Thirumazhisai, Kathavur and Pallavedu, Veppampattu, Mittinamallee, Vilinjiyambakkam, Periyar Nagar, Parithipattu, Papparambakkam, Ulundai, and Poonamallee. It was made sure that all the selected PHCs are comparable with regard to available resources and living conditions. This ensured that these PHCs are not systematically different from each other and are representative of all the PHCs in Chennai.

The study plans to recruit a total of 25,000 individuals using a nonprobability complete enumeration sampling method. The selected sampling method would allow the researchers to study more than one aspect of all items of the population and obtain data from each and every unit of population. Each item will be observed personally by the researchers. The collected data will be reliable, accurate, and truly representative of the whole population. In addition, the data obtained using the complete enumeration method can be used as a basis for future studies [22]. The eligible study participants will comprise the following: individuals 18 years and older, individuals residing in the selected urban and rural settings, and individuals consenting to participate in the study. Individuals having any mental or physical challenges that might affect their ability to participate in the study will be excluded from the study.

### Informed Consent

The institutional review board–approved informed consent form will be administered by the research team to the eligible individuals for the study. The research team will describe the study, time required, and benefits of the study results to the participants, and those willing to participate and give their consent will be enrolled in the study. The participants should be able to read and understand the questionnaire. In case any participant is illiterate, ethical consent will be obtained with the help of a legally acceptable representative or an impartial witness [23]. In addition, the illiterate participants will be explained the questionnaire in the local Indian dialects to aid in the usefulness and generalizability of the study. Data gathered will be stored in a secure manner ensuring data privacy and confidentiality. Written informed consent will be obtained in both English and local Indian dialects. Study participants will be allowed to withdraw from the study at any time mentioning the reasons for withdrawal. All data including those from study withdrawals will be reported in the final analysis. No monetary compensation will be given, and those agreeing to participate will be offered snack meals. Every individual’s time will be respected, and their voluntary participation will be appreciated. Nonmonetary forms of compensation would help to avoid coercion and undue inducement that might impact the results of the study [24].

### Data Collection, Data Entry, and Quality Assurance

Data collection and data entry will be performed by a team of data collectors and data management personnel. The data will be collected at a one-time point by administering the study questionnaire to the eligible study participants (Multimedia Appendix 1). For designing and standardizing the questionnaire, the researchers performed a pilot study involving a sample of 2500 individuals. Initial data will be gathered on paper and then entered into the computer using Microsoft Excel (Microsoft Corporation). For the main survey, the data will be recorded electronically using computer-based software. To ensure efficiency and high-quality data collection and processing, the following data management protocol is in place: a clearly defined study manual, a well-trained team of data collectors, weekly meetings with the research team, weekly data checks, maintenance of study participant contact, and maintenance of study participant data instrument logs.

### Variable Assessment

#### Sociodemographic Profile

This data will be gathered on study participants’ age, gender, income level, education level, employment status, occupation, region of residence, marital status, parenthood, and religion.

#### Health Status Profile

This will include data on comorbidities, health insurance, COVID-19 diagnostic tests, and anthropometry measurements such as height and weight using a standard technique. The two measurements will aid in calculating the BMI of study participants.

#### Prior Immunization

This would include questions related to experiences with previous seasonal, influenza, and COVID-19 immunizations.

#### History of COVID-19

This comprises questions related to individuals and their family members’ history of COVID-19.

#### Knowledge, Attitude, and Practices Related to COVID-19

This data gathers participant’s COVID-19 knowledge levels and their attitude and practices toward preventive practices to minimize the spread of COVID-19. The information recorded will help to design targeted public health messaging to address knowledge, attitude, and practices related to COVID-19.

#### Knowledge, Attitude, and Barriers Related to COVID-19 Vaccination

This data would gather participant’s knowledge, attitudes, and barriers related to COVID-19 vaccination so that appropriate public health messaging can be established to enhance uptake of COVID-19 vaccination and follow through on safe, preventive COVID-19–related practices.

#### COVID-19 Vaccine Acceptance and Hesitancy

Data on individuals’ preferences related to the COVID-19 vaccine will be gathered.

#### Communication and Misinformation About the COVID-19 Pandemic and Vaccination

This data records the sources of COVID-19 information. Additionally, data on the use of protective measures against the infection will be gathered.

#### Unintended Consequences of COVID-19

This records data related to the lifestyle and behavioral changes including impact on mental health. Information related to mobile and internet use as sources of COVID-19 information will also be gathered. Generalized anxiety disorder will be assessed using the Generalized Anxiety Disorder 7 self-administered patient questionnaire [25], while anxiety as a result of COVID-19 will be assessed using a 7-item COVID-19 anxiety scale [26].

### Data Security and Privacy

Data security will be ensured through regular backups, password-protected computers, and data files stored in a locked file cabinet in an office. The information will be accessible to members of the research team only. Data will be stored in a password-protected computer in a locked office of the principal investigator for 3 years from the point of study completion at which time they will be destroyed.

### Outcomes

The study outcomes include factors associated with COVID-19 vaccine acceptance and hesitancy, and knowledge, attitude, and practices related to COVID-19 and vaccination. The authors of the study will also explore the factors affecting unintended consequences of COVID-19 infection across urban and rural settings in an Indian setting.

### Data Analysis Plan

The gathered data will be presented in tables comprising the recorded characteristics of all variables. These tables will serve the purpose of data quality control to find out inconsistencies in the data patterns and outliers or any missing data. Descriptive analysis will be conducted to report the means and SDs of the continuous variables and frequency analysis of the categorical variables. *T* tests will be performed to compare the means between the continuous variables and a categorical dependent variable, while chi-square analysis will be performed for the categorical variables. Multivariate regression analysis will be performed to determine the predictors of the outcome variables of vaccine acceptance and hesitancy. All analysis will be performed using SAS v9.1 and reporting of the results will be done at 95% CIs and *P*=.049.

### Project Timeline and Milestones

A detailed research plan and scheduled timeline of the tasks involved in the study are presented in Table 1.

**Table 1:** Scheduled timeline of the tasks in the CO-VIN-CAP study.

Task	Month											
	1	2	3	4	5	6	7	8	9	10	11	12-24
Review of the literature, initial designing, and planning of the study	✓^a^	—^b^	—	—	—	—	—	—	—	—	—	—
Development of study proposal and ethical approval	✓	—	—	—	—	—	—	—	—	—	—	—
Approval of the study proposal	✓	—	—	—	—	—	—	—	—	—	—	—
Development of survey items and the questionnaire	✓	—	—	—	—	—	—	—	—	—	—	—
Review and revision of the questionnaire by the research team	—	✓	—	—	—	—	—	—	—	—	—	—
Recruitment and training of the data collector team	—	✓	—	—	—	—	—	—	—	—	—	—
Pilot testing of the representative sample of the target population	—	—	✓	✓	—	—	—	—	—	—	—	—
Initial data analysis, results write up, and dissemination of the pilot survey	—	—	—	—	—	✓	✓	—	—	—	—	—
Revision of the questionnaire based on the pilot testing	—	—	—	✓	—	—	—	—	—	—	—	—
Development of electronic survey	—	—	—	✓	—	—	—	—	—	—	—	—
Recruitment of the target sample	—	—	—	—	✓	✓	✓	✓	✓	✓	✓	—
Reviewing collected data by the research team	—	—	—	—	✓	✓	✓	✓	✓	✓	✓	✓
Data analysis	—	—	—	—	—	—	✓	✓	✓	✓	✓	✓
Results write up and preparation of the manuscript	—	—	—	—	—	—	—	✓	✓	✓	✓	✓
Dissemination	—	—	—	—	—	—	—	✓	✓	✓	✓	✓

^a^Indicates it will be done in this month.

^b^Indicates it will not be done in this month.

### Ethics and Dissemination

The study bearing protocol number PMCHRI-IHEC-029 gained approval from the Panimalar Medical College Hospital and Research Institute Institutional Human Ethics Committee (Central Drugs Standard Control Organization Registration No. ECR/1399/Inst/TN/2020) in January 2021 with approval No. PMCH&RI/IHEC/2021/037 dated January 13, 2021. The study will be conducted according to the Declaration of Helsinki, as it involves human participants [27].

Findings of the study will be disseminated through peer-reviewed publications and national and international conference presentations. Findings will also be disseminated to the local community health leaders and other state officials and policy makers for data-driven, evidence-based informed decision making.

## Results

The proposed research study will help explore the burden of vaccine acceptance and hesitancy among individuals living in urban and rural settings of Chennai, Tamil Nadu. Further, it will help to examine the variables that influence vaccine acceptance and hesitancy. The data collection was initiated on March 1, 2021, and the initial results are planned for publication by June 2021. The result findings of the study will help to design and develop a user-centered informatics platform that can deliver multimedia-driven health educational modules tailored to facilitate vaccine uptake in varied settings.

## Discussion

The study will provide insights toward the barriers and challenges leading to lower vaccine acceptance rates. The research would help in identifying the key areas that need to be addressed through intervention to enhance the compliance of COVID-19 vaccine acceptance. There is a need for strategies to increase vaccine literacy and to directly address community-specific misconceptions regarding vaccines and at the same time be sensitive to religious or philosophical beliefs. The survey would help in assessing the rates of vaccine acceptance and hesitancy and its determinants among the population of Chennai, Tamil Nadu. The findings from this research project would help in identifying, developing, and implementing data-driven, evidence-based, and human-centered behavior modification interventions to address COVID-19 vaccine hesitancy among populations living in diverse settings.

The study would provide an in-depth understanding of various factors related to COVID-19 vaccine acceptance, intention, and hesitancy among individuals living in urban and rural Indian settings. The results of the study may be used to conduct a statistical comparison with similar studies to test and evaluate similarities or differences in the outcomes across diverse settings nationally and internationally. It would help the researchers of the study to formulate appropriate interventions. However, further research involving long follow-up is needed to explore the impact of such interventions on long-term outcomes.

## Appendix

Multimedia Appendix 1 Full text of the survey questionnaire.

## Abbreviations

NFHS: National Family Health Survey
PHC: primary health center
WHO: World Health Organization
