# Supplementary material for: The Impact of COVID-19 Vaccine Communication, Acceptance, and Practices (CO-VIN-CAP) on Vaccine Hesitancy in an Indian Setting: Protocol for a Cross-sectional Study
Source: JMIR Res Protoc. 2021 Jun 24;10(6):e29733. doi: 10.2196/29733 (PMC8386355; doi:10.2196/29733)
Supplement: Multimedia Appendix 1 [file resprot_v10i6e29733_app1.docx]

**Multimedia Appendix 1.** Full text of the self-administered questionnaire.

**SURVEY CATEGORIES**

1. Data Collector Information
2. Geographical information
3. Individual Profile
4. Socio-demographic profile
5. Health status profile
6. Prior immunization
7. History of COVID-19 disease
8. Knowledge, attitudes, and practices related to COVID-19 disease
9. Knowledge, attitudes, and barriers related to COVID-19 vaccination
10. COVID-19 vaccine acceptance and hesitancy
11. Communication and misinformation about COVID-19 pandemic and vaccination
12. Unintended consequences of COVID-19
13. **DATA COLLECTOR INFORMATION**

**A1. Date of visit** dd-mm-yyyy

**A2. Time of visit** _ _:_ _ hours

**A3. Names of Data Collection team**

**A4. Mode of travel (to and from the location)**

1. Vehicle provided by team/responsible organisation
2. Public transport
3. Walk
4. Other, please specify_____
5. **GEOGRAPHICAL INFORMATION**

**B1. PHC name-**

**B2. Pin code-**

**B3. Location and coordinates-**

**B4. Distance from the PMCHRI (in m/km)-**

1. **INDIVIDUAL PROFILE**

**C1. Name of respondent:**

**C2. Respondent ID:**

**C3. Occupation:**

**C4. PHC:**

**C5. SOCIO-DEMOGRAPHIC**

**C5a. Age group (in years)**

1. 18-24
2. 25-54
3. 55-64
4. 65+

**C5b. Gender**

1. Male
2. Female
3. Other, please specify _________

**C5c. Education Level**

1. Illiterate
2. Primary school certification
3. Middle School certification
4. High school certification
5. Intermediate or diploma
6. Graduate
7. Profession or Honours

**C5d. Monthly Income level**

1. ≤10,001
2. 10,002-29,972
3. 29,973-49,961
4. 49,962-74,755
5. 74,756-99,930
6. 99,931-199,861
7. ≥199,862

**C5e. Marital Status**

1. Unmarried
2. Married
3. Divorced/Separated
4. Widowed

**C5f. Religion**

1. Hindu
2. Sikh
3. Christian
4. Muslim
5. Other, please specify___________
6. Not willing to tell

**C5g. Region of Residence**

1. Urban
2. Rural
3. Slum

**C5h. Employment Status**

1. Unemployed
2. Employed/Self-employed
3. Retired
4. Homemaker
5. Lost job due to COVID-19

**C5i. Occupation**

1. Unemployed
2. Elementary Occupation
3. Plant and Machine Operators and Assemblers
4. Migrant labourer
5. Craft and Related Trade Workers
6. Skilled Workers and Shop and Market Sales Workers
7. Skilled Agricultural and Fishery Workers
8. Clerks
9. Technicians and Associate Professionals
10. Professionals
11. Legislators, Senior Officials and Managers
12. Frontline worker/Healthcare worker, please specify _________

**C5j. Do you have a child/children?**

1. Yes
2. No

C5ja. If yes, number of children, please specify _________

C5jb. Age of each child, please specify _________

1. **HEALTH STATUS PROFILE**

**D1. Do you have any underlying physician confirmed illnesses?**

1. Yes
2. No
3. I do not know

D1a. If yes, please specify________

**D2. Are you enrolled into any health insurance program?**

1. No
2. Private
3. Government

**D2a.** If government insurance, please specify the name of the insurance scheme you are enrolled in_________

**D3. Have you been tested for COVID-19?**

1. Yes
2. No
3. Not willing to tell

D3a. If yes, what was the result of your COVID-19 test?

1. Positive
2. Negative
3. Not willing to tell

D3b. If the COVID-19 test result was positive, how severe were your symptoms?

1. No symptoms
2. Mild Symptoms
3. Moderate symptoms but did not seek help from a doctor
4. Moderate symptoms and sought help from a doctor
5. Severe symptoms and was hospitalized

D3c. If the COVID-19 test result was positive, did you practice any home remedy to treat the infection?

1. Yes
2. No
3. Not willing to tell

D3d. If yes, please specify which home remedy you practiced? ________

**D4. Height (in cm)**

**D5. Weight (in kg)**

**D6. Body mass index (BMI) (kg/m2)**

1. **PRIOR IMMUNIZATION**

**E1. In the past, have you ever refused to take a vaccine?**

1. Yes
2. No
3. Not willing to tell

**E2. In the past, have you ever had a bad reaction to a vaccine?**

1. Yes
2. No
3. Not sure
4. Not willing to tell

E2a. If yes, please specify the event_____________________________

**E3. Do you know anyone who has had a bad reaction to a vaccine previously?**

1. Yes
2. No
3. Not sure
4. Not willing to tell

**E4. Would the vaccination related bad events discourage you from taking a COVID-19 vaccine?**

1. Yes
2. No
3. Not Sure
4. Not willing to tell

**E5. Have you received a COVID-19 vaccine?**

1. Received one dose of vaccine
2. Received two doses of vaccine
3. No

E5a. If you received the first dose of COVID-19 vaccine, do you plan to take the second dose of it?

1. Yes
2. No
3. Not sure

E5b. If no, please specify the reason _________

1. **HISTORY OF COVID-19 DISEASE**

**F1. Do you know anyone in your household or immediate family who has contracted COVID-19 infection?**

1. Yes
2. No
3. Not willing to tell

F1a. If yes, please specify who had contracted COVID-19 infection? ________________

F1b. If yes, how severe were their symptoms of the COVID-19 infection?

1. No symptoms
2. Mild Symptoms
3. Moderate symptoms but did not seek help from a doctor
4. Moderate symptoms and seeked help from a doctor
5. Severe symptoms and was hospitalized
6. Resulted in death
7. **KNOWLEDGE, ATTITUDES, AND PRACTICES RELATED TO COVID-19 DISEASE**

**GK1- COVID-19 is an infectious disease.**

1. True
2. False
3. Not sure

**GK2- Fever, dry cough, sore throat, diarrhoea, and shortness of breath are the main symptoms of COVID-19 infection.**

1. True
2. False
3. Not sure

**GK3- Individuals who have COVID-19 infection but do not show any symptoms can transmit the infection.**

1. True
2. False
3. Not sure

**GK4- COVID-19 infection can spread via the respiratory droplets during coughing/sneezing by the infected person in the air.**

- - - 1. True
      2. False
      3. Not sure

**GK5- Wearing facemask when going out is an effective way to prevent contracting or spreading the COVID-19 infection.**

1. True
2. False
3. Not sure

**GK6- Washing hands with soap and water for sanitizing the hands with alcohol based sanitizer after touching any object/handshaking/coughing/sneezing is an effective way to prevent contracting or spreading the COVID-19 infection?**

1. True
2. False
3. Not sure

**GK7 - After how many days do the symptoms of COVID-19, appear in an infected person?**

1. 2-5 days
2. 2-14 days
3. 5-10 days

**GA1- In your opinion, how serious do you believe the COVID-19 infection is?**

1. Not at all serious
2. A little serious
3. Somewhat serious
4. Quite a bit serious
5. Very serious

**GA2- In your opinion, correct health education regarding COVID-19 can prevent the spread of COVID-19 infection.**

1. True
2. False
3. Not sure

**GA3- Perceived risk of COVID-19 infection - How likely do you think it is that you will contract COVID-19 infection?**

1. Very High
2. High
3. Fair
4. Low
5. Very low
6. No perceived risk

**GA4- In your opinion, closure of schools and universities was an appropriate measure taken by the government to prevent the COVID-19 infection?**

1. Yes
2. No
3. Not Sure

**GA5- In your opinion, closure of workplaces and offices was an appropriate measure taken by the government to prevent the COVID-19 infection?**

1. Yes
2. No
3. Not sure

**GA6- In your opinion, is COVID-19 virus of natural or man-made origin?**

1. Natural
2. Man-made
3. Not sure

**GP1- Do you wear a facemask when you go out from your home?**

1. Always
2. No
3. Sometimes

**GP2- Do you wash your hands with soap and water or sanitize them frequently, especially whenever you touch something/cough/sneeze?**

1. Always
2. No
3. Sometimes

**GP3- Do you practice social distancing (staying 6 feet apart from the other person) when outside?**

1. Always
2. No
3. Sometimes

**GP4- Do you avoid unnecessary travelling or going out for recreational activities in order to prevent contracting or spreading the infection?**

1. Always
2. No
3. Sometimes

**GP5- Do you avoid touching your face and eyes especially when in public places?**

1. Always
2. No
3. Sometimes

**GP6- Do you cover your mouth/nose when you cough or sneeze with handkerchief/tissue?**

1. Yes always
2. No
3. Sometimes

**GP7- Do you avoid consuming outside food in order to** **prevent contracting or spreading the infection?**

1. Yes always
2. No
3. Sometimes

**GP8- Do you follow the COVID-19 guidelines issued by your state or central government authorities?**

1. Yes always
2. No
3. Sometimes

**GP9 - After the pandemic, would you limit your contact such as handshaking with other people?**

1. Yes
2. No
3. Sometimes

**GP10- Do you disinfect or sanitize the touched surfaces or objects?**

1. Yes
2. No
3. Sometimes
4. **KNOWLEDGE, ATTITUDES, AND BARRIERS RELATED TO COVID-19 VACCINATION**

**HK1- Currently there are no vaccines available in India to protect against COVID-19 infection.**

1. True
2. False
3. Not Sure

**HK2- Do you think vaccines strengthen the immune system?**

1. Yes
2. No
3. Not sure

**HK3- Does one need to follow preventive measures such as wearing a mask, hand sanitization, social distancing after receiving the COVID 19 vaccine?**

1. True
2. False
3. Not Sure

**HK4-** **How many doses of vaccine would have to be taken by a person and at what interval?**

1. Two doses of vaccine with 28 days apart
2. One dose of vaccine with 28 days apart
3. Two doses of vaccine with 14 days apart
4. One dose of vaccine with 14 days apart
5. I do not know

**HK5- COVID-19 vaccine is mandatory by the government.**

1. True
2. False
3. Not Sure

**HK6- When would antibodies develop after getting a COVID-19 vaccine?**

1. Two weeks after receiving the second dose of COVID-19 vaccine
2. One week after receiving the second dose of COVID-19 vaccine
3. Two weeks after receiving the first dose of COVID-19 vaccine
4. One week after receiving the first dose of COVID-19 vaccine
5. I do not know

**HA1- In your opinion, taking a COVID-19 vaccine is important for preventing COVID-19 infection.**

1. True
2. False
3. Not Sure

**HA2- How would you describe your attitude towards receiving a COVID-19 vaccine?**

1. Very interested
2. Somewhat interested
3. Neutral/no opinion
4. Somewhat interested
5. Not at all interested

**HA3- Would you encourage your family and friends to take a COVID-19 vaccine?**

1. Yes
2. No
3. Not sure

**HA4- If the vaccine is developed outside India, would it make you more willing to receive it?**

1. Yes
2. No
3. I do not know

**HA5- It is important for everyone to get a COVID-19 vaccine once available.**

1. Completely agree
2. Somewhat agree
3. Neutral/no opinion
4. Somewhat disagree
5. Completely disagree

**HA6- In your opinion, COVID-19 vaccination should be mandatory for everyone once available.**

1. Completely agree
2. Somewhat agree
3. Neutral/no opinion
4. Somewhat disagree
5. Completely disagree

**HA7- In your opinion, do you believe that vaccine producers are interested in your health?**

1. Yes
2. No
3. I do not know

**HA8- In your opinion, do you trust vaccine manufacturers on safety and effectiveness of a COVID-19 vaccine?**

1. Yes
2. No
3. Not sure

**HA9- What is the maximum amount of time you would be willing to spend to get a COVID-19 vaccine once available?**

1. None
2. 0 to 30 min
3. 30 to 60 min
4. 60 to 90 min
5. 90 to 120 min
6. 120 min and over

**HA10- How likely are you willing to take the two-dose recommended schedule of a COVID-19 vaccine?**

1. Extremely likely
2. Very likely
3. Somewhat likely
4. Not very likely
5. Not at all likely

**HA11- The side effects of a COVID-19 vaccine would be more than its benefits.**

1. Strongly agree
2. Agree
3. Uncertain
4. Disagree
5. Strongly disagree

**HA12- In your opinion, do you think a COVID-19 vaccine would cause severe side effects?**

1. Yes
2. No
3. Not sure

**HA13- Do you perceive that a COVID-19 vaccine would be beneficial to your health?**

1. Yes
2. No
3. Not sure

**HB1- Do you feel that the doctor’s recommendation is an important factor while deciding to get a COVID-19 vaccine?**

1. Completely agree
2. Somewhat agree
3. Neutral/no opinion
4. Somewhat disagree
5. Completely disagree

**HB2- Which type of COVID-19 vaccine would you prefer?**

1. Vaccine manufactured in India
2. Vaccine manufactured outside India
3. Any vaccine
4. None

**HB3- What would be your preferred mode of vaccine administration once available to the public?**

1. Orally
2. Injected
3. Nasal spray
4. None of the above

**HB4- Would the cost of COVID-19 vaccine prevent you from getting vaccinated if it was not provided for free?**

1. Yes
2. No
3. Not sure

**HB5- What is the maximum amount of money (in Rupees) you would be willing to pay for a COVID-19 vaccine once available?**

1. None
2. 10-100 INR.
3. 101-200 INR.
4. 201-300 INR.
5. >301 INR.

**HB6- Would you take a vaccine if it is provided free of cost?**

1. Yes
2. No
3. Not sure

**HB7- If you have to spend a lot of time travelling to get COVID-19 vaccination, would you consider it important enough to travel for it?**

1. Yes
2. No
3. Not sure

**HB8- Do you feel that the fast-paced COVID-19 vaccine development will fail to detect potential side effects?**

1. Strongly agree
2. Agree
3. Uncertain
4. Disagree
5. Strongly disagree
6. **COVID-19 VACCINE ACCEPTANCE AND HESITANCY**

**I1. Would you accept a COVID-19 vaccine if available?**

1. Yes
2. May be yes (VH)
3. Not sure (VH)
4. May be not (VH)
5. No (Reject)

**I2. Would you accept a COVID-19 vaccine if available for free?**

1. Yes
2. May be yes (VH)
3. Not sure (VH)
4. May be not (VH)
5. No (Reject)

**I3. Would you accept a COVID-19 vaccine if it is 50% effective?**

1. Yes
2. May be yes (VH)
3. Not sure (VH)
4. May be not (VH)
5. No (Reject)

**I4. Would you accept a COVID-19 vaccine if it is 70% effective?**

1. Yes
2. May be yes (VH)
3. Not sure (VH)
4. May be not (VH)
5. No (Reject)

**I5. Would you accept a COVID-19 vaccine with 90-95% efficacy?**

1. Yes
2. May be yes (VH)
3. Not sure (VH)
4. May be not (VH)
5. No (Reject)

I5a. If yes, specify the reason for vaccinating yourself against COVID-19 infection ______________________

I5b. If maybe yes/Not sure/Maybe not, specify the reasons for having hesitancy towards getting yourself vaccinated_______________________________

I5c. If no, specify the reason for not vaccinating yourself against COVID-19 infection ___________________________________________

**I6. Would you accept a COVID-19 vaccine if your employer recommended it?**

1. Yes
2. May be
3. Not Sure
4. May be not
5. No

**I7. Would you accept a COVID-19 vaccine if your doctor recommended it?**

1. Yes
2. May be
3. Not Sure
4. May be not
5. No

**I8. What would motivate you to accept a COVID-19 vaccine? Please specify ______________________**

**I9. Would you accept a COVID-19 vaccine for children when it is available?**

1. Yes
2. May be
3. Not Sure
4. May be not
5. No

I9a. If yes, specify the reason for vaccinating your child/children vaccinated against COVID-19 infection ______________________

I9b. If maybe yes/Not sure/Maybe not , specify the reasons for having hesitancy towards getting your child/children vaccinated against COVID-19 infection _______________________________

I9c. If no, specify the reason for not vaccinating your child/children against COVID-19 infection ___________________________________________

**I10. Would you accept a COVID-19 vaccine if successfully developed and approved?**

1. Yes
2. May be
3. Not Sure
4. May be not
5. No
6. **COMMUNICATION AND MISINFORMATION ABOUT COVID-19 PANDEMIC AND VACCINATION**

**J1. Which is your primary source of COVID-19 infection and vaccine information? (select all those applicable)**

1. News channels
2. Radio channels
3. Television
4. Government websites
5. Newspaper/magazine
6. Healthcare workers (community health worker/primary health worker)
7. Family and friends
8. Internet
9. Social media
10. WHO/CDC website
11. Any other, please specify______

**J2. What are the sources of information you have the most confidence and trust in when it comes to seeking COVID-19 disease and vaccine related information? Select all those applicable**

1. News channels
2. Radio channels
3. Government websites
4. Newspaper/Magazines
5. Healthcare workers (community health worker/primary health worker)
6. Family and friends
7. Internet
8. Social media
9. WHO/CDC website
10. Any other, please specify______

**J3. How frequently do you watch/read/listen to the news about the COVID-19?**

1. Not at all
2. Once a week
3. 3-4 times a week
4. Once a day
5. 2-3 times a day
6. More than 3 times a day
7. Almost all day

**J4. Do you think vitamins and mineral supplements can cure COVID-19 infection?**

1. True
2. False
3. Not sure

**J5. Do you trust the government’s response to deal effectively with the COVID-19 pandemic?**

1. Not at all
2. Not much
3. Neutral
4. Partially trusted
5. Can be trusted fully

**J6. Do you think the prolonged use of surgical masks when properly worn causes carbon dioxide intoxication or oxygen deficiency?**

1. True
2. False
3. I do not know

## J7. Do you think Spraying and introducing bleach or another disinfectant into your body will protect you against COVID-19 infection?

1. Yes
2. No
3. Maybe

## J8. Does regular rinsing the nose with saline prevent COVID-19 infection?

1. Yes
2. No
3. May be
4. **MISINFORMATION ABOUT COVID-19 VACCINE (Questions Prepared from myths buster - WHO/CDC)**

**K1. Do you think a COVID-19 vaccine can make you sick with COVID-19 infection?**

1. Yes
2. No
3. I do not know

## K2. Do you think that a COVID-19 vaccine can affect women and men’s fertility?

1. Yes
2. No
3. I do not know

**K3. Do you think a COVID-19 will alter your DNA?**

1. Yes
2. No
3. I do not know

**K4. Do you think that once vaccinated with COVID-19 vaccine you can stop wearing a mask?**

1. Yes
2. No
3. I do not know

**K5. Do you think a second dose of a vaccine is not important?**

1. Yes
2. No
3. I do not know
4. **UNINTENDED CONSEQUENCES OF COVID-19**

**L1. How would you describe your personal life during COVID-19?**

1. Very Challenging
2. Somewhat challenging
3. Similar/Neutral
4. Somewhat less challenging
5. Not at all challenging

**L2. How would you describe your social life during COVID-19?**

1. Very challenging
2. Somewhat challenging
3. Similar/Neutral
4. Somewhat less challenging
5. Not at all challenging

**L3. How would you describe your professional life during COVID-19?**

1. Very challenging
2. Somewhat challenging
3. Similar/Neutral
4. Somewhat less challenging
5. Not at all challenging

**L4. How has COVID-19 affected your relationships with family members?**

1. Significantly improved
2. Slightly improved
3. No change
4. Slightly decreased
5. Significantly decreased

**L5. Has your daily tasks changed since the pandemic? (Work, school, social, leisure, and religion related activities or other ways you normally spend your time)**

1. No changes to daily tasks
2. Mild changes to daily tasks
3. Moderate changes to daily tasks
4. Severe changes to daily tasks

**L6. How have your day-to-day resource needs suffered during COVID-19?**

1. Severely affected
2. Moderately affected
3. Mildly affected
4. Not at all affected
5. Improved

**L7. How has COVID-19 affected your employment?**

1. Became unemployed
2. Similar
3. Became employed

**L8. How has COVID-19 affected your quality of work?**

1. Severely affected
2. Moderately affected
3. Mildly affected
4. Not at all affected
5. Improved

**L9. How has COVID-19 affected your income?**

1. Greatly reduced
2. Moderately reduced
3. Not at all affected
4. Moderately increased
5. Greatly increased

**L10. How has COVID-19 affected your child’s education?**

1. Severely affected
2. Moderately affected
3. Mildly affected
4. Not at all affected
5. Improved

**L11. Did you have to move from your current residence because of COVID-19?**

1. Yes
2. No

L11a. If yes, please specify reason ______

**Lifestyle and behavioural changes**

**L12. How has your physical activity (exercising, brisk walking, jogging, jumping) changed during COVID-19?**

1. No, I do not normally exercise
2. No, I have been exercising with the same frequency and intensity as you usually do
3. Yes, I have been exercising regularly, but with less intensity than usual
4. Yes, I have not been exercising as regularly as usual, but the intensity is the same as usual
5. Yes, I have not been exercising at all and very sedentary.

**L13. How have your sleeping hours changed during COVID-19?**

1. Significantly increased
2. Slightly increased
3. Grossly similar
4. Slightly decreased
5. Significantly decreased

**L14. How has your quality of sleep changed during COVID-19?**

1. Much better
2. Somewhat better
3. Similar/No change
4. Somewhat worse
5. Much worse

**L15. How has your screen time (social media/television/mobile phone) changed during COVID-19?**

1. Significantly increased
2. Slightly increased
3. Grossly similar
4. Slightly decreased
5. Significantly decreased

**L16. How has your consumption of unhealthy foods (salty snacks, carbonated drinks, fried snacks, chocolates) changed during COVID-19?**

1. Significantly increased
2. Slightly increased
3. Grossly similar
4. Slightly decreased
5. Significantly decreased

**L17. How has your habit of snacking changed during COVID-19?**

1. Significantly increased
2. Slightly increased
3. Grossly similar
4. Slightly decreased
5. Significantly decreased

**L18. How has your fruits and vegetables intake changed during COVID-19?**

1. Significantly increased
2. Slightly increased
3. Grossly similar
4. Slightly decreased
5. Significantly decreased

**L19. How has your tobacco use (smoking and smokeless tobacco) changed during COVID-19?**

1. Significantly increased
2. Slightly increased
3. Grossly similar
4. Slightly decreased
5. Significantly decreased
6. Not willing to tell

**L20. How has your alcohol use changed during COVID-19?**

1. Significantly increased
2. Slightly increased
3. Grossly similar
4. Slightly decreased
5. Significantly decreased
6. Not willing to tell

**L21. How has COVID-19 affected your child’s health?**

1. Severely affected
2. Moderately affected
3. Mildly affected
4. Not at all affected
5. Improved

**Impact on mental health**

**L22. How has COVID-19 affected your mental health?**

1. Much better
2. Somewhat better
3. Similar/No change
4. Somewhat worse
5. Much worse

**L23. How has COVID-19 affected your child’s mental health due to closure of schools?**

1. Severely affected
2. Moderately affected
3. Mildly affected
4. Not at all affected
5. Improved

**L24. How has mental health been affected due to the financial strain caused by COVID-19?**

1. Severely affected
2. Moderately affected
3. Mildly affected
4. Not at all affected

**L25. Have you had a death due to COVID-19 or any other reason in your family?**

1. Yes
2. No
3. Not willing to tell

L25a. If yes, please specify how it has affected your mental health?________________________

1. **Generalized Anxiety - Source -** <https://patient.info/doctor/generalised-anxiety-disorder-assessment-gad-7>

| **S.No** | **Statement** | **Over the last 2 weeks, how often have you been bothered by any of the following problems?** | | | |
| --- | --- | --- | --- | --- | --- |
|  |  | **1 = Not at all** | **2 = Several days** | **3 = More than half the days** | **4 =Nearly every day** |
| M1. | Feeling nervous, anxious or on edge |  |  |  |  |
| M2. | Not being able to stop or control worrying |  |  |  |  |
| M3. | Worrying too much about different things |  |  |  |  |
| M4. | Trouble relaxing |  |  |  |  |
| M5. | Being so restless that it is hard to sit still |  |  |  |  |
| M6. | Becoming easily annoyed or irritable |  |  |  |  |
| M7. | Feeling afraid as if something awful might happen |  |  |  |  |

1. **COVID-19 - Anxiety Scale - To which extent do the following statements apply to you right now? Source -** [**https://www.ncbi.nlm.nih.gov/pmc/articles/PMC7661558/**](https://www.ncbi.nlm.nih.gov/pmc/articles/PMC7661558/)

| **S.No** | **Statement** | **How much has each item reflected your behavior in the last days? Please, indicate using the scale.** | | | |
| --- | --- | --- | --- | --- | --- |
|  |  | **0 = Not applicable to me** | **1 = Rarely applicable to me** | **2 = Sometimes applicable to me** | **3 = Very applicable to me** |
| N1. | I feel bad when thinking about COVID-19 |  |  |  |  |
| N2. | I feel heart racing when I read about COVID-19 |  |  |  |  |
| N3. | I feel anxious about COVID-19 |  |  |  |  |
| N4. | I feel uneasy when reading news about COVID-19 |  |  |  |  |
| N5. | I have trouble relaxing when I think about COVID-19 |  |  |  |  |
| N6. | I feel like I may panic when I update myself about COVID-19 |  |  |  |  |
| N7. | I am afraid of being infected with COVID-19 |  |  |  |  |
